# Supplementary material for: Topical Application of Antrodia cinnamomea Ointment in Diabetic Wound Healing
Source: Life (Basel). 2022 Mar 30;12(4):507. doi: 10.3390/life12040507 (PMC9027955; doi:10.3390/life12040507)
Supplement: Supplementary file 1 [file life-12-00507-s001.zip › life-1596922-supplementary.pdf]

Supplementary Materials

5×10[4] RAW264.7 cells were treated with medium alone, 0.1% DMSO/0.001% ethanol (vehicle), LPS (0.1 µg/mL), LPS (0.1 µg/mL) plus IFN-γ (5U), or various doses of AC extracts (50, 100, 200, 400, 800 µg/mL) as indicated. Culture supernatants were collected after 24 h. The amounts of nitrite were measured by Griess assay. Data are expressed as mean of nitrite (µM) ± SD of four independent experiments.

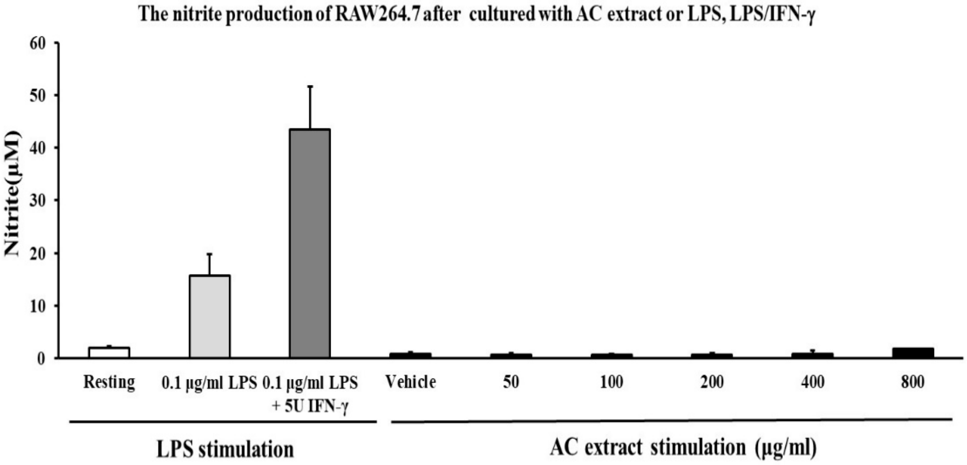

Figure S1. Addition of the AC extract alone did not activate macrophages.

5×10[4] RAW264.7 cells were treated with 0.1% DMSO/0.001% ethanol (vehicle) or various doses of AC extracts (50, 100, 200, 400, 800 µg/mL) as indicated. After 24 h incubation, the proliferation of RAW264.7 cells was measured by MTT assay. Data are expressed as mean of OD570 nm ± SD of four independent experiments.

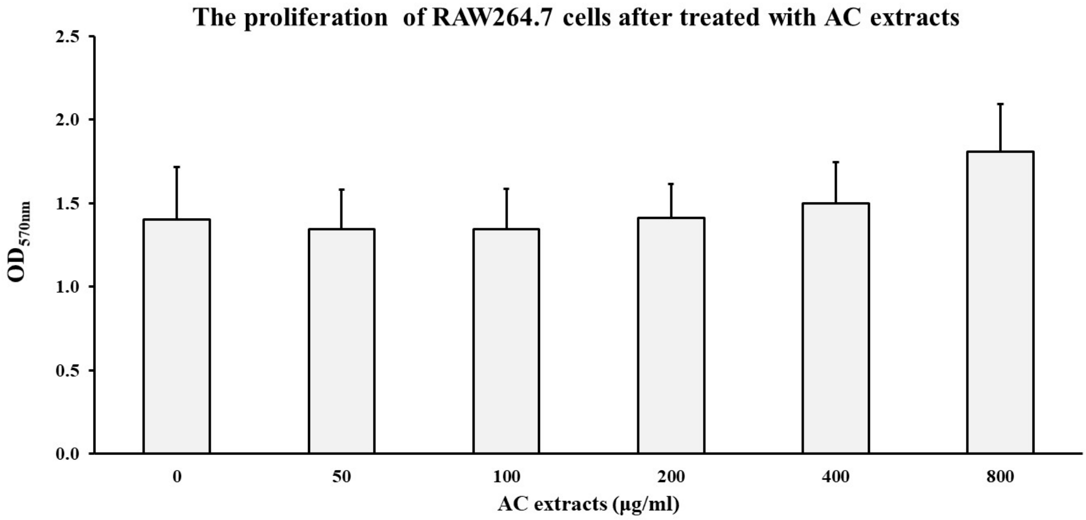

Figure S2. The effect of AC extracts on the proliferation of RAW 264.7 cell

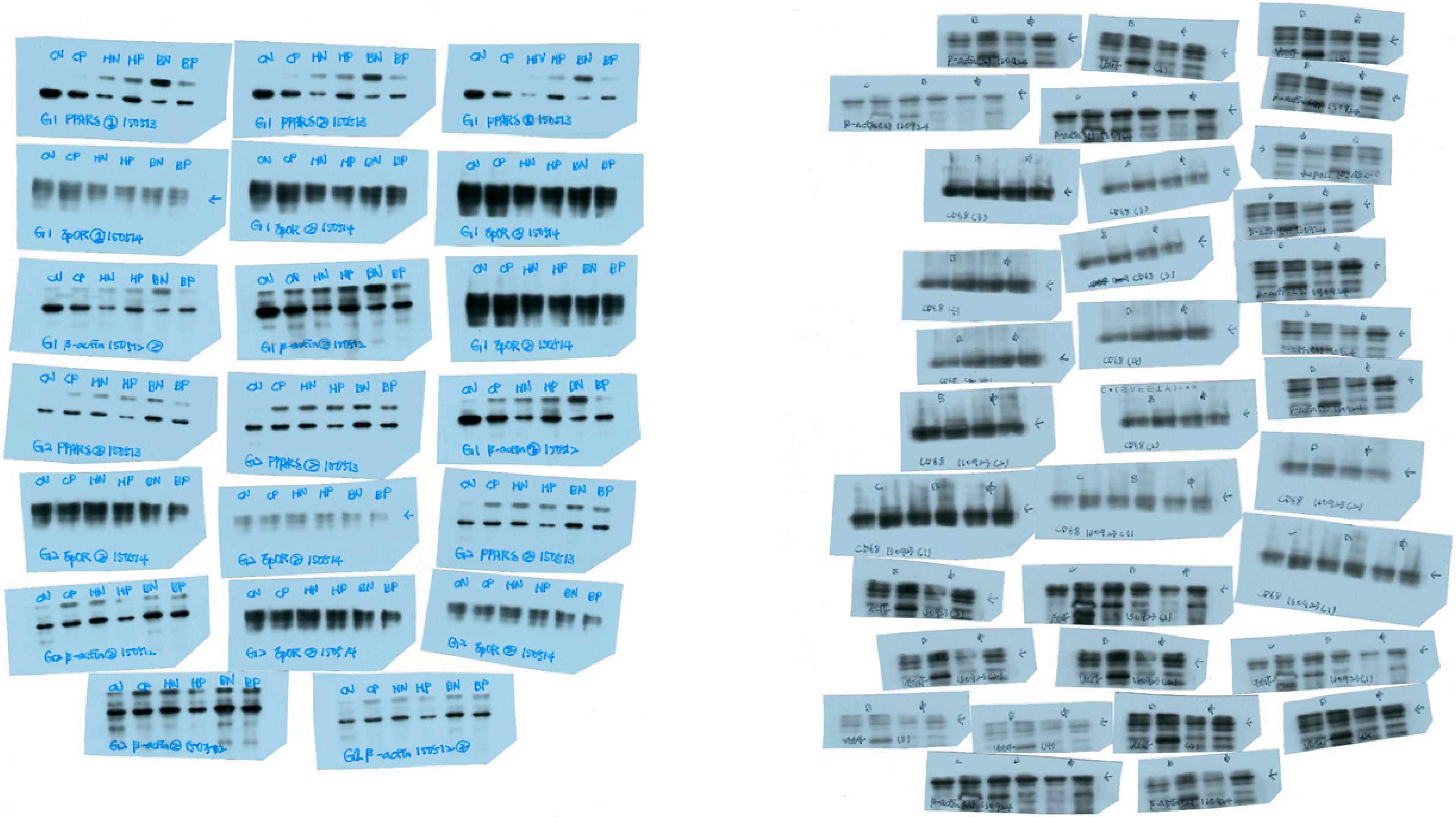

Figure S3. The original Western blot figures.
